# Supplementary material for: StemPanTox: A fast and wide-target drug assessment system for tailor-made safety evaluations using personalized iPS cells
Source: iScience. 2022 Jun 6;25(7):104538. doi: 10.1016/j.isci.2022.104538 (PMC9218511; doi:10.1016/j.isci.2022.104538)
Supplement: Document S1. Figures S1–S8 and Table S1–S3 and S5–S9 [file mmc1.pdf]

iScience, Volume *25*

Supplemental information

StemPanTox: A fast and wide-target drug assessment system for tailor-made safety evaluations using personalized iPS cells

Junko Yamane, Takumi Wada, Hironori Otsuki, Koji Inomata, Mutsumi Suzuki, Tomoka Hisaki, Shuichi Sekine, Hirokazu Kouzuki, Kenta Kobayashi, Hideko Sone, Jun K. Yamashita, Mitsujiro Osawa, Megumu K. Saito, and Wataru Fujibuchi

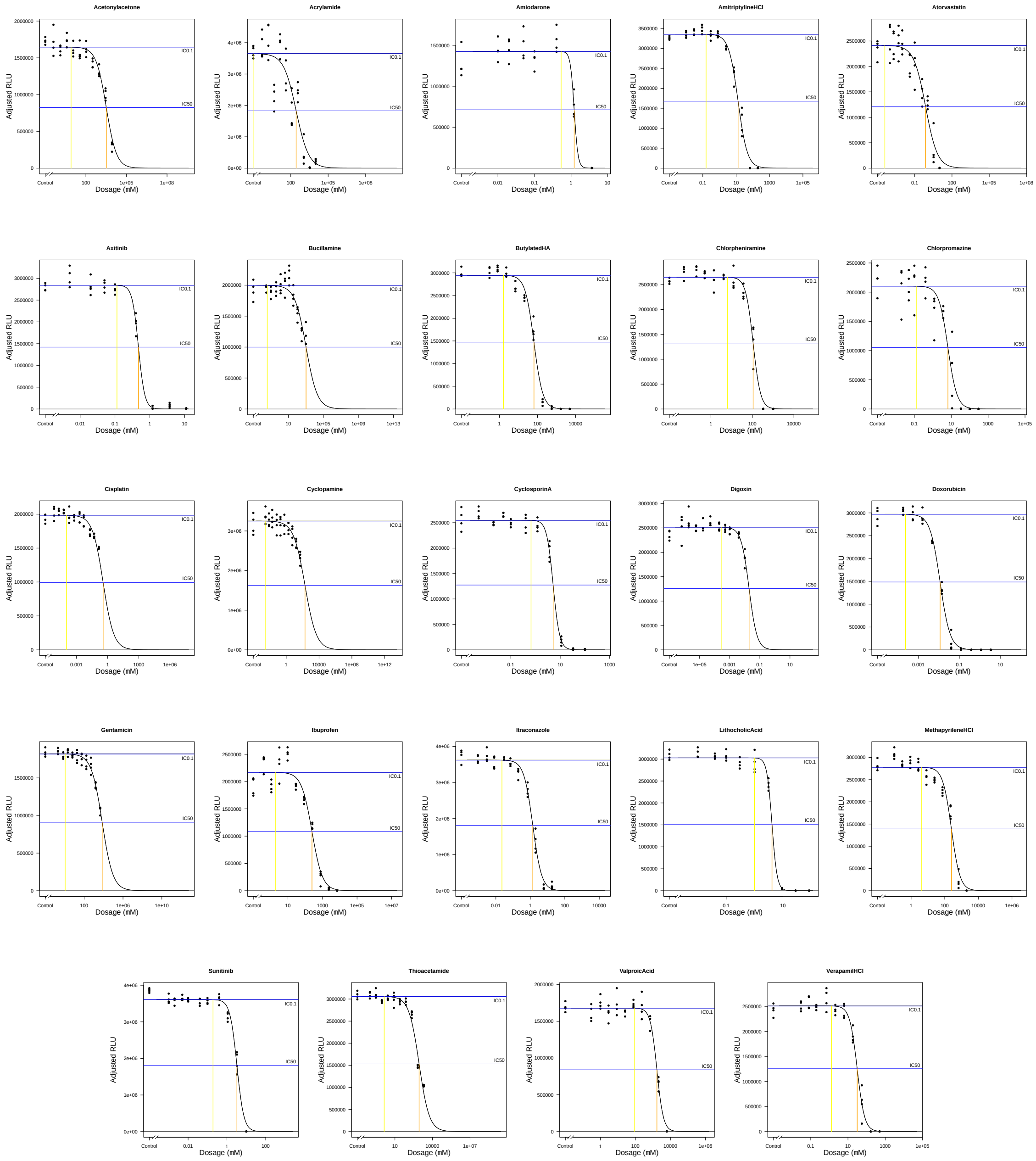

Figure S1. Dose response curves of the 24 chemicals in the ATP assay using KhES-3 cells, related to Figure 1

A stock solution was prepared with the highest soluble concentration. To determine the exposure doses for testing, we performed 10 serial three-fold dilutions of the stock solution. The ATP assay was performed 48 h after exposure. Regression analysis was performed by fitting the three-parameter log-logistic model with the drc package in R statistical language to determine IC0.1 and IC50 values.

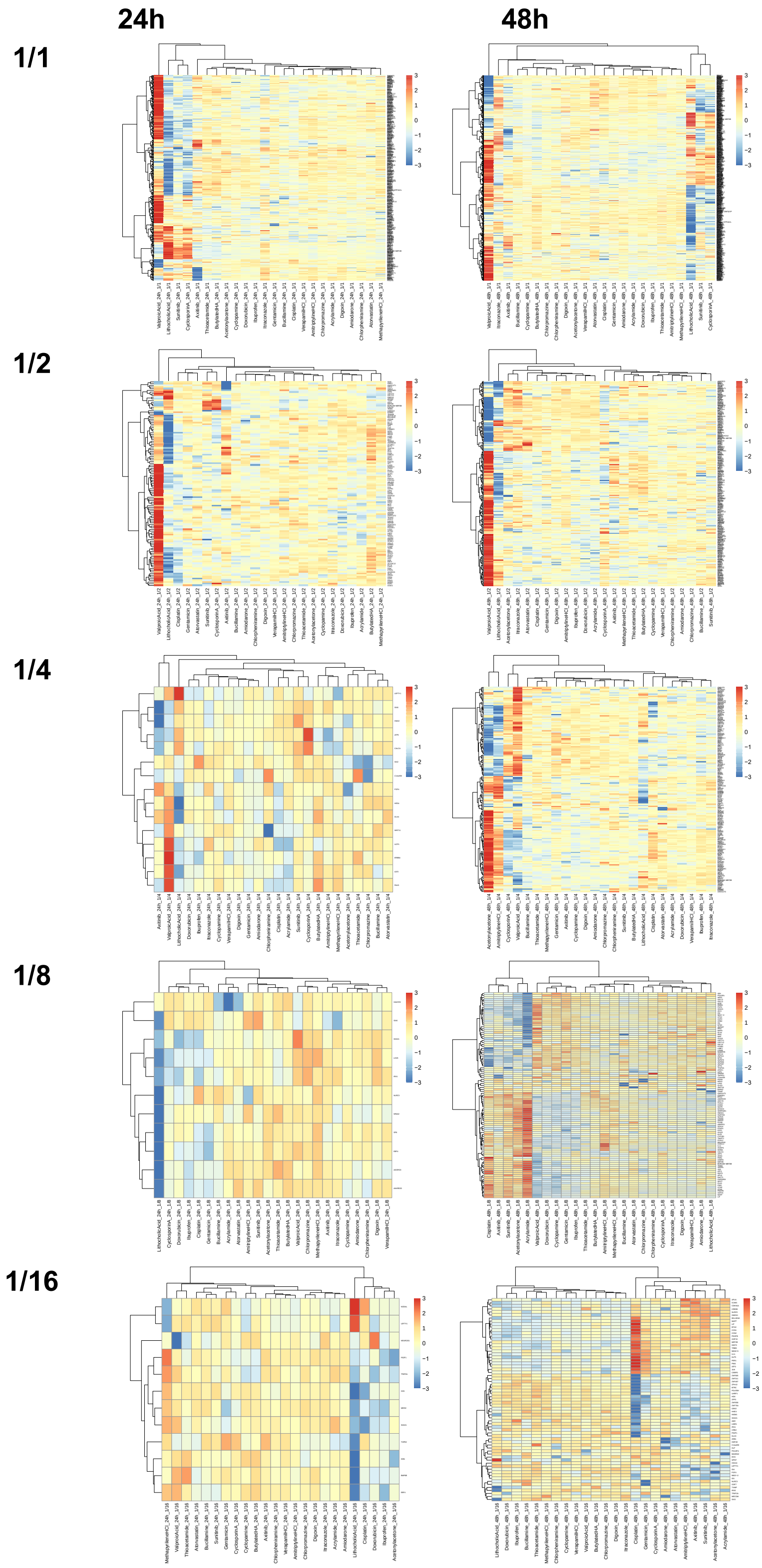

Figure S2. Differentially expressed transcription factor genes for 24 chemicals at five doses at 24 h and 48 h, related to Figure 1

The exposure dose was set between IC0.1 and IC50 depending on the degree of cell death as the maximum exposure dose, and five serial two-fold dilutions (1/1, 1/2, 1/4, 1/8, 1/16) and solvent-only exposure were performed. We analyzed differentially expressed genes (DEGs) by the log-fold–change (LFC) between the 24 chemicals and their corresponding solvent, and created a heatmap of genes with LFC > 1 and FDR (false discovery rate) < 0.01.

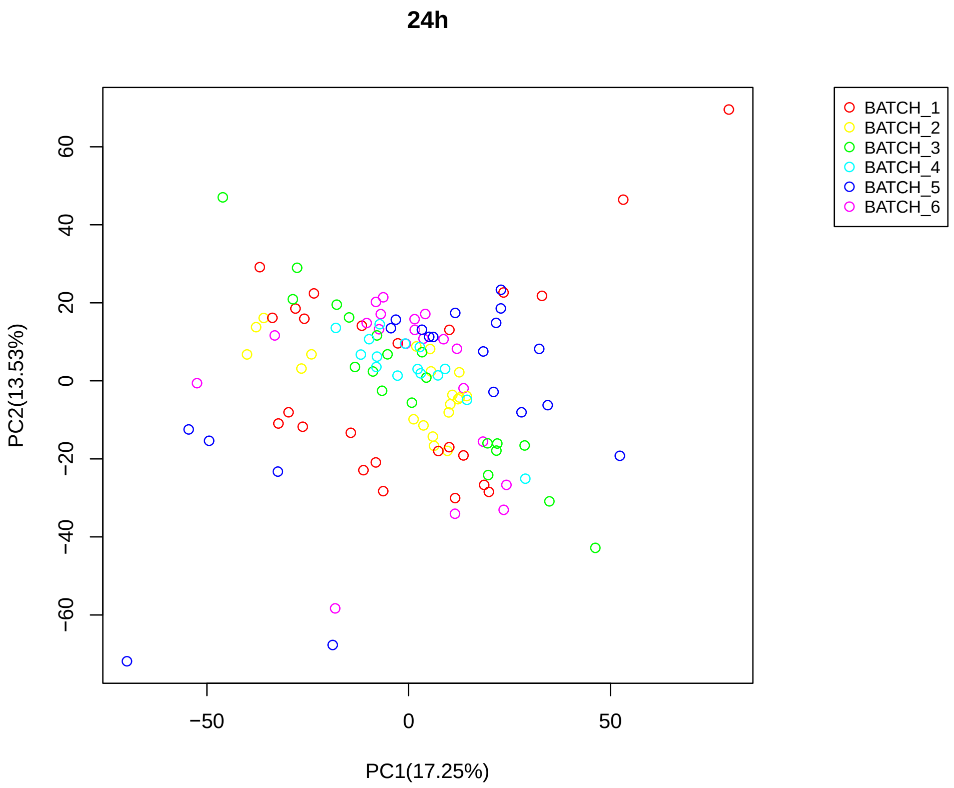

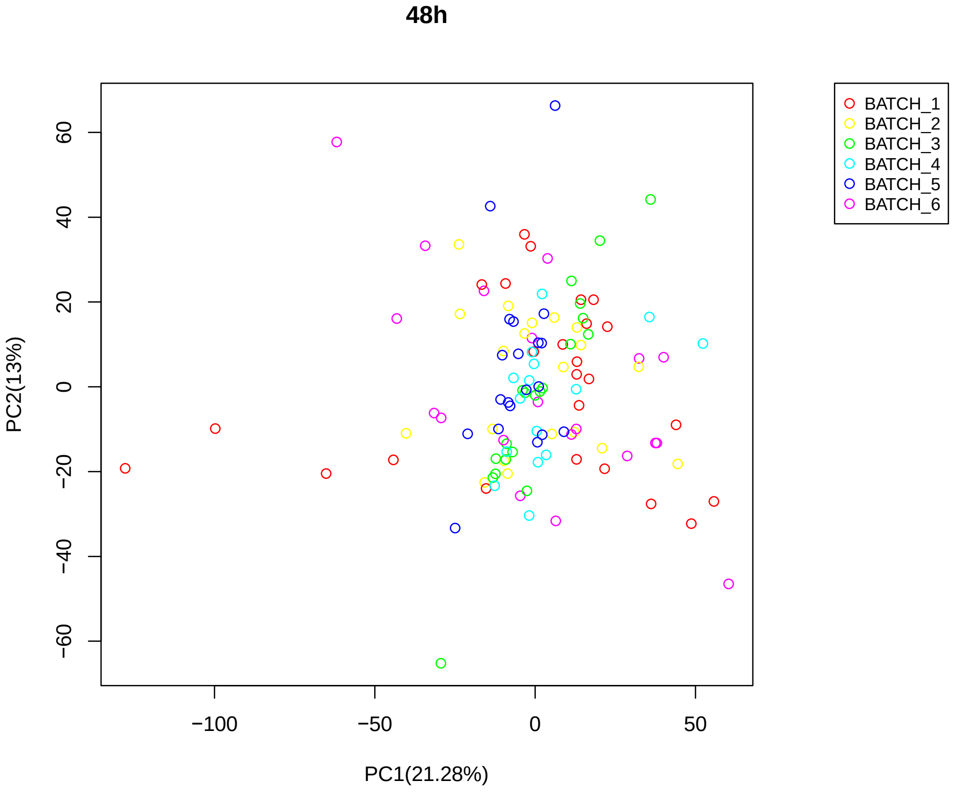

Figure S3. PCA analysis of KhES-3 cell data at 24 h and 48 h, related to Figure 1

Using the log-fold–change values obtained for 120 (24 chemicals x 5 doses) conditions, PCA was performed for each of the two time points (24 h, 48 h). The removeBatchEffect function in the package edgeR was used to eliminate batch effects.

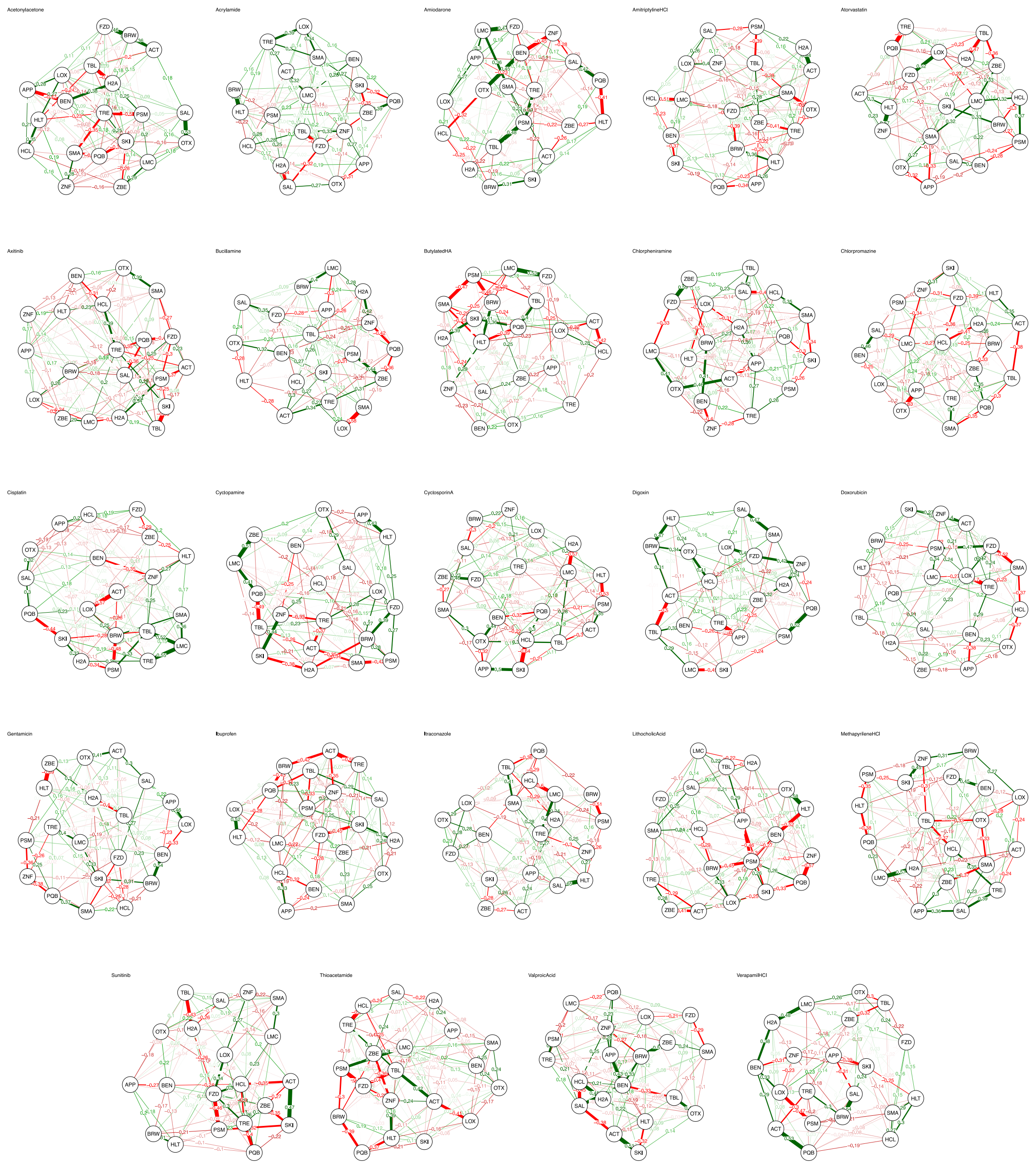

Figure S4. GGM for each of the 24 chemicals at 24 h, related to Figure 2

On the basis of the results of the PCA, a total of 20 genes (two genes each with the top positive and negative loading values in the first to fifth PCs) were used to construct gene networks. To estimate GGM for each of the 24 chemicals, we used the aforementioned log-fold–change values to calculate the sparse partial correlation coefficient network with L1 graphical lasso using EBICglasso in the R package qgraph.

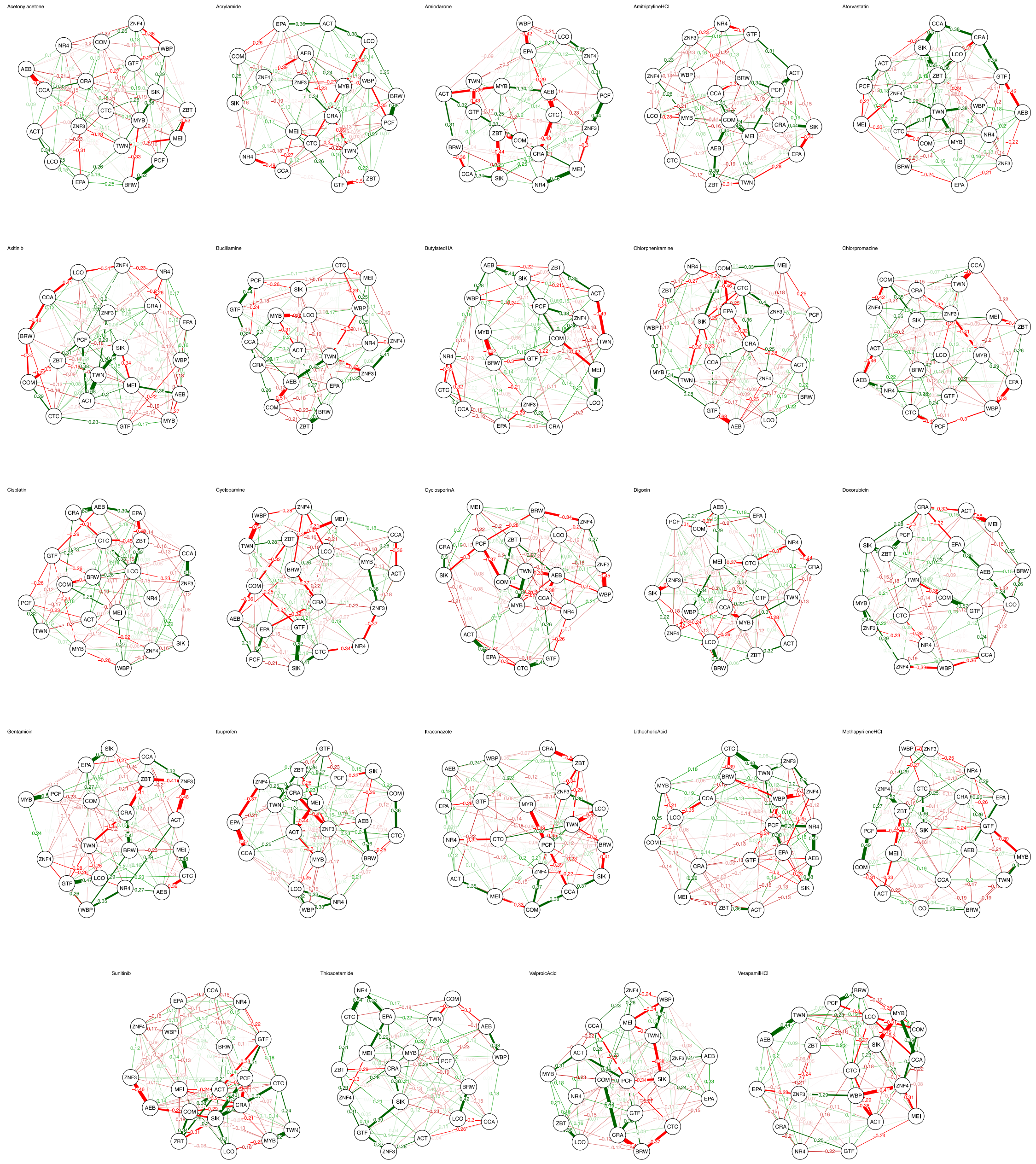

Figure S5. GGM for each of the 24 chemicals at 48 h, related to Figure 2

Please refer to Figure S4. legend.

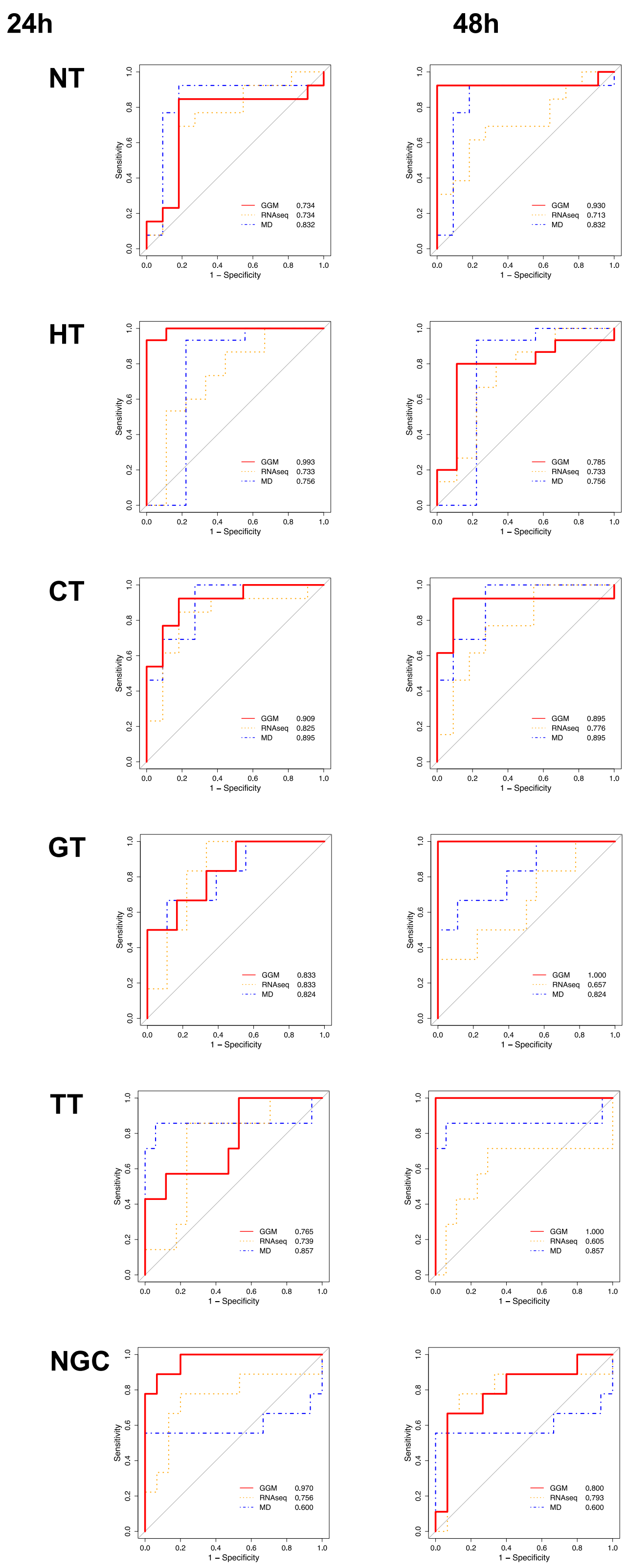

Figure S6. ROC curves for predictions of 6 toxic categories at 24 h and 48 h, related to Figure 2

We used AUC (area under the receiver operating characteristic curve) for the statistical measurement of prediction performance. GGM, Graphical Gaussian Model by 20 selected transcription factor genes; RNAseq, log-fold–change values from RNA-seq data of 3,200 (24 h) and 3,255 (48 h) transcription factor genes; MD, 5,666 molecular descriptors for the 24 chemicals. NT, Neurotoxin; HT, Hepatotoxin; CT, Cardiotoxin; GT, Glomerular toxin (Nephrotoxin); TT, Tubular toxin (Nephrotoxin); NGC, Non-genotoxic carcinogen.

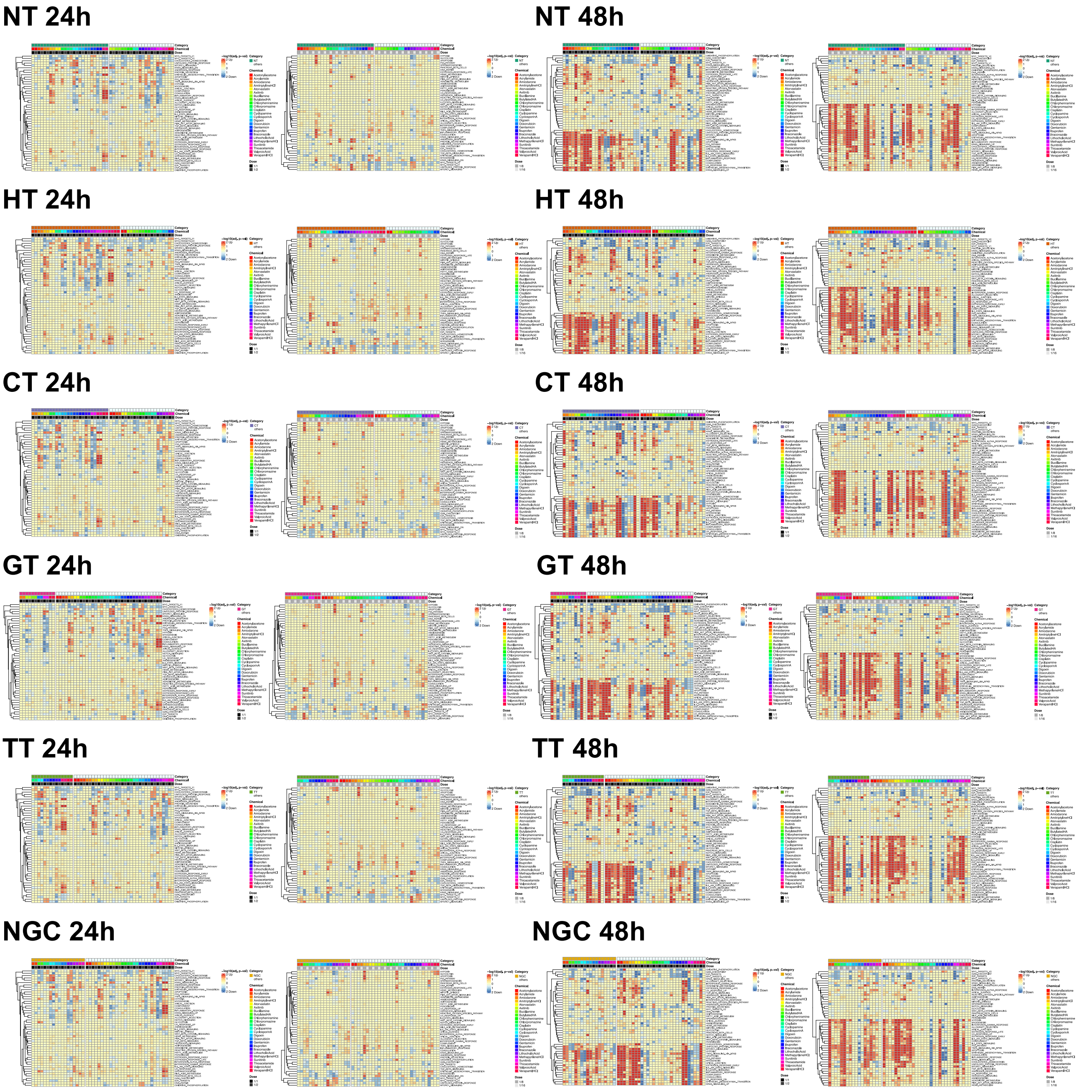

left: high doses

right: low doses

Figure S7. Pathway analysis by GSEA of the 6 toxic categories, related to Figure 2

The log-fold–change values for all genes (21,650 and 22,298 genes for 24 h and 48 h, respectively) were divided into high-dose (1/1/, 1/2) and low-dose (1/8, 1/16) groups to perform GSEA analysis for 50 hallmark gene sets. A heatmap was generated with FDR-adjusted p values obtained using the fgsea package in R. NT, Neurotoxin; HT, Hepatotoxin; CT, Cardiotoxin; GT, Glomerular toxin (Nephrotoxin); TT, Tubular toxin (Nephrotoxin); NGC, Non-genotoxic carcinogen.

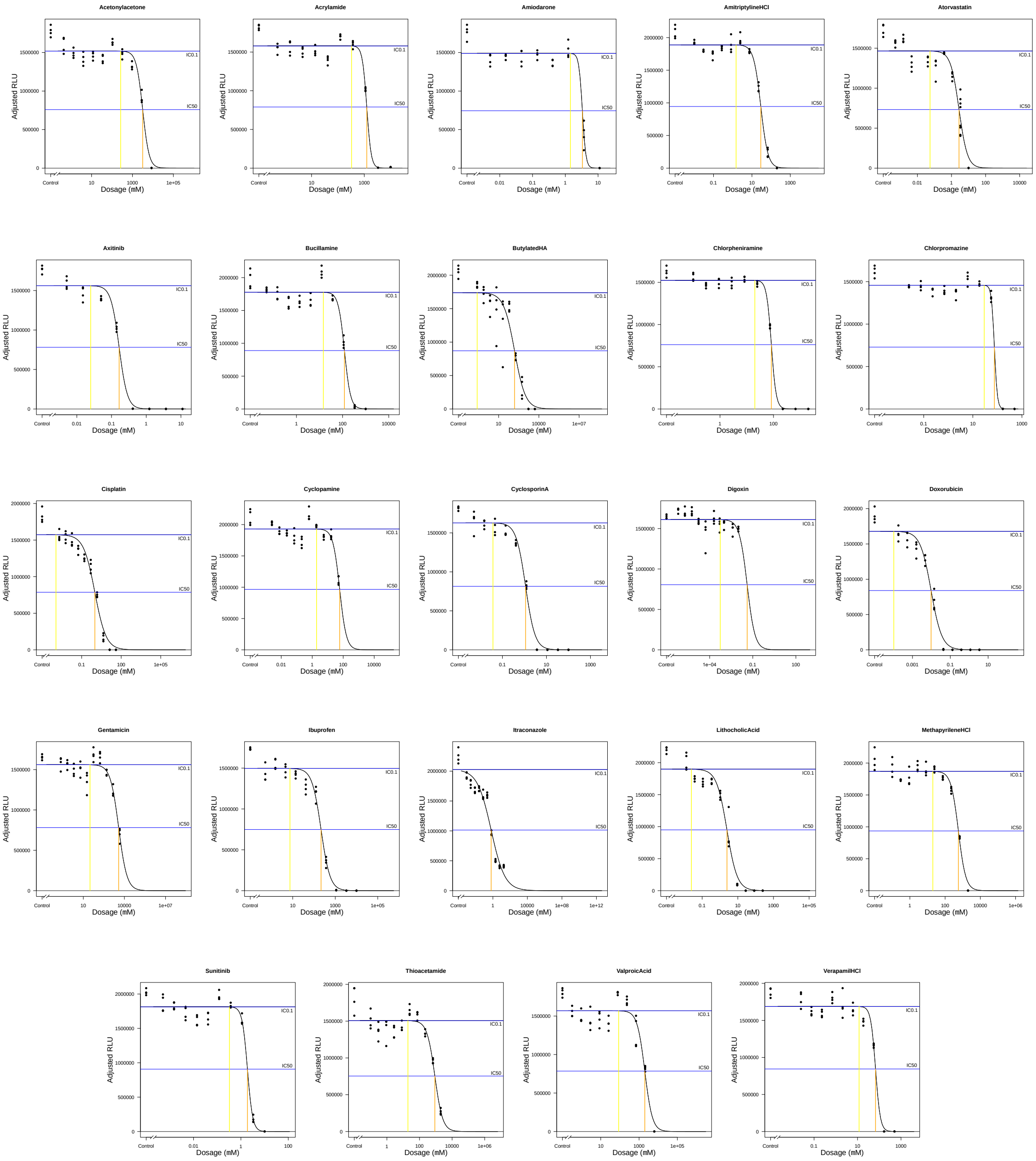

Figure S8. Dose response curves of 24 chemicals in the ATP assay using HPS4138 cells, related to Figure 3

Please refer to Figure S1. legend.

Chemical name	Solvent	MaxSolv (ES)	IC0.1	IC50	IC99.9	Exposure 1/1	Exposure 1/2	Exposure 1/4	Exposure 1/8	Exposure 1/16
Acetonylacetone	DMSO	8525	7.977377	3237.53	1313916	100	50	25	12.5	6.25
Acrylamide	DMSO	10000	0.09560427	271.5933	771544.1	100	50	25	12.5	6.25
Amiodarone	DMSO	100	0.5343475	1.218097	2.77677	0.99	0.495	0.2475	0.12375	0.061875
AmitriptylineHCl	DMSO	200	0.1571875	13.02059	1078.557	3.2	1.6	0.8	0.4	0.2
Atorvastatin	DMSO	10	3.91E-04	0.7525803	1448.366	0.05	0.025	0.0125	0.00625	0.003125
Axitinib	DMSO	100	0.1139036	0.4718385	1.954561	0.3	0.15	0.075	0.0375	0.01875
Bucillamine	DMSO	1000	0.03860087	1051.014	28616703	50	25	12.5	6.25	3.125
ButylatedHA	DMSO	5000	1.661853	65.19734	2557.804	20	10	5	2.5	1.25
Chlorpheniramine	DMSO	1000	6.365731	108.5013	1849.36	40.5	20.25	10.125	5.0625	2.53125
Chlorpromazine	DMSO	300	0.1342938	6.614745	325.8145	1.78	0.89	0.445	0.2225	0.11125
Cisplatin	Water	40/0.14	1.09E-04	0.3677969	1236.932	0.01	0.005	0.0025	0.00125	0.000625
Cyclopamine	DMSO	50	0.00320417	194.7581	11837913	50	25	12.5	6.25	3.125
CyclosporinA	DMSO	100	0.6602198	5.176834	40.59195	2.7	1.35	0.675	0.3375	0.16875
Digoxin	DMSO	250/0.01	2.99E-04	0.01934455	1.251801	0.01	0.005	0.0025	0.00125	0.000625
Doxorubicin	DMSO	3.45	2.34E-04	0.01146006	0.5603627	0.0003	0.00015	0.000075	0.0000375	0.00001875
Gentamicin	Water	4500	1.102839	7198.079	46980892	500	250	125	62.5	31.25
Ibuprofen	DMSO	7000	1.958154	246.4163	31009.31	3	1.5	0.75	0.375	0.1875
Itraconazole	DMSO	18	0.02258895	1.387967	85.28298	0.56	0.28	0.14	0.07	0.035
LithocholicAcid	DMSO	250	0.9948314	4.103289	16.92446	2.6	1.3	0.65	0.325	0.1625
MethapyrileneHCl	Water	2000	4.280212	250.9152	14709.18	47.2	23.6	11.8	5.9	2.95
Sunitinib	DMSO	10	0.1933371	3.29441	56.13582	1.3	0.65	0.325	0.1625	0.08125
Thioacetamide	Water	2000	1.388053	860.3128	533220.4	198	99	49.5	24.75	12.375
ValproicAcid	DMSO	6240	90.74899	1684.708	31275.73	500	250	125	62.5	31.25
VerapamilHCl	DMSO	500	1.330277	31.23197	733.258	11.2	5.6	2.8	1.4	0.7

Table S1. ATP assay data and exposure concentrations (uM) of KhES3, related to Figure 1

Chemical name	Estimate	Std. Error	Lower	Upper
Acetonylacetone	3237.53	300.7016	2630.251	3844.81
Acrylamide	271.5933	92.97792	83.82057	459.366
Amiodarone	1.218097	0.04206249	1.13315	1.303044
AmitriptylineHCl	13.02059	0.7064965	11.59379	14.44739
Atorvastatin	0.7525803	0.1472552	0.4551925	1.049968
Axitinib	0.4718385	0.0365324	0.3980599	0.5456172
Bucillamine	1051.014	213.9036	619.0265	1483.001
ButylatedHA	65.19734	4.043097	57.03215	73.36254
Chlorpheniramine	108.5013	6.171486	96.0377	120.9648
Chlorpromazine	6.614745	1.012063	4.570843	8.658647
Cisplatin	0.3677969	0.05026668	0.2662813	0.4693125
Cyclopamine	194.7581	63.2097	67.10349	322.4127
CyclosporinA	5.176834	0.2250185	4.722399	5.631268
Digoxin	0.01934455	0.00521373	0.0088152	0.02987389
Doxorubicin	0.01146006	0.00050039	0.0104495	0.01247061
Gentamicin	7198.079	776.8786	5629.141	8767.017
Ibuprofen	246.4163	31.13065	183.5467	309.2859
Itraconazole	1.387967	0.07413565	1.238247	1.537687
LithocholicAcid	4.103289	0.2376307	3.623384	4.583194
MethapyrileneHCl	250.9152	23.87428	202.7001	299.1303
Sunitinib	3.29441	0.1348621	3.02205	3.566769
Thioacetamide	860.3128	55.32658	748.5785	972.0471
ValproicAcid	1684.708	93.34396	1496.196	1873.22
VerapamilHCl	31.23197	1.728015	27.74218	34.72177

Table S2. IC50 concentrations (uM) and 95 % confidence intervals of KhES3, related to Figure 1

Time	Gene weight	Ensemble ID	Gene name	Description
24h	0.039190508	ENSG00000071794	HLTF	Helicase like transcription factor
	0.036623731	ENSG00000165288	BRWD3	Bromodomain and WD repeat domain containing 3
	-0.038051915	ENSG00000102103	PQBP1	Polyglutamine binding protein 1
	-0.038175834	ENSG00000159377	PSMB4	Proteasome 20S subunit beta 4
	0.037541037	ENSG00000214717	ZBED1	Zinc finger BED-type containing 1
	0.037291576	ENSG00000134013	LOXL2	Lysyl oxidase like 2
	-0.041978467	ENSG00000115091	ACTR3	Actin related protein 3
	-0.043192873	ENSG00000136603	SKIL	SKI like proto-oncogene
	0.04883801	ENSG00000180340	FZD2	Frizzled class receptor 2
	0.04784571	ENSG00000124496	TRERF1	Transcriptional regulating factor 1
	-0.046221333	ENSG00000080503	SMARCA2	SWI/SNF related, matrix associated, actin dependent regulator of chromatin, subfamily a, member 2
	-0.047444721	ENSG00000071282	LMCD1	LIM and cysteine rich domains 1
	0.058446399	ENSG00000178409	BEND3	BEN domain containing 3
	0.055188868	ENSG00000165588	OTX2	Orthodenticle homeobox 2
	-0.048981141	ENSG00000142192	APP	Amyloid beta precursor protein
	-0.051095983	ENSG00000180353	HCLS1	Hematopoietic cell-specific Lyn substrate 1
	0.060630288	ENSG00000180573	H2AC6	H2A clustered histone 6
	0.052387905	ENSG00000203326	ZNF525	Zinc finger protein 525
	-0.052967868	ENSG00000101849	TBL1X	Transducin beta like 1 X-linked
	-0.055815075	ENSG00000103449	SALL1	Spalt like transcription factor 1
48h	0.036044803	ENSG00000123358	NR4A1	Nuclear receptor subfamily 4 group A member 1
	0.035739786	ENSG00000132471	WBP2	WW domain binding protein 2
	-0.0355102	ENSG00000197937	ZNF347	Zinc finger protein 347
	-0.035561226	ENSG00000198464	ZNF480	Zinc finger protein 480
	0.040199463	ENSG00000132382	MYBBP1A	MYB binding protein 1a
	0.039495166	ENSG00000158941	CCAR2	Cell cycle and apoptosis regulator 2
	-0.044333891	ENSG00000185658	BRWD1	Bromodomain and WD repeat domain containing 1
	-0.045006616	ENSG00000178177	LCORL	Ligand dependent nuclear receptor corepressor like
	0.048845727	ENSG00000115091	ACTR3	Actin related protein 3
	0.045999961	ENSG00000181472	ZBTB2	Zinc finger and BTB domain containing 2
	-0.046930046	ENSG00000105419	MEIS3	Meis homeobox 3
	-0.047639865	ENSG00000143320	CRABP2	Cellular retinoic acid binding protein 2
	0.046575675	ENSG00000155115	GTF3C6	General transcription factor IIIC subunit 6
	0.04656354	ENSG00000102974	CTCF	CCCTC-binding factor
	-0.049551424	ENSG00000116016	EPAS1	Endothelial PAS domain protein 1
	-0.04961551	ENSG00000106624	AEBP1	AE binding protein 1
	0.048918291	ENSG00000149600	COMMD7	COMM domain containing 7
	0.048279472	ENSG00000142178	SIK1	Salt inducible kinase 1
	-0.053231857	ENSG00000165494	PCF11	PCF11 cleavage and polyadenylation factor subunit
	-0.054237905	ENSG00000107815	TWNK	Twinkle mtDNA helicase

Table S3. List of feature genes for 6 categories by PCA, related to Figure 1

Chemical name	24 h	48 h
Acetonylacetone	100	100
Acrylamide	83.3	100
Amiodarone	83.3	83.3
AmitriptylineHCl	100	83.3
Atorvastatin	100	100
Axitinib	66.6	66.6
Bucillamine	100	83.3
ButylatedHA	83.3	83.3
Chlorpheniramine	100	83.3
Chlorpromazine	100	83.3
Cisplatin	66.6	66.6
Cyclopamine	83.3	50
CyclosporinA	66.6	66.6
Digoxin	100	100
Doxorubicin	83.3	100
Gentamicin	83.3	100
Ibuprofen	66.6	83.3
Itraconazole	100	100
LithocholicAcid	100	100
MethapyrileneHCl	100	83.3
Sunitinib	66.6	100
Thioacetamide	100	83.3
ValproicAcid	83.3	100
VerapamilHCl	50	83.3

Table S5. The accuracy of 6 toxicity category predictions for each chemical (%), related to Figure 2

Chemicals	KhES-3	HPS4138	HPS4234	HPS4046
Acetonylacetone	0.828978495	0.754764297	0.671117415	0.377693978
Acrylamide	1.036990388	1.07742624	0.998743795	0.890617915
Amiodarone	0.813959931	0.883409701	0.694363962	0.226528875
AmitriptylineHCl	1.017463864	0.882575463	0.942187553	1.018290015
Atorvastatin	0.794666576	0.874481107	0.865066018	0.579273968
Bucillamine	1.050059887	0.9180794	0.969580927	1.070701767
ButylatedHA	0.995743146	0.870512843	0.218910614	0.446486392
Chlorpheniramine	0.242997415	0.000854279	0.003511971	0.000373915
Chlorpromazine	0.558634478	0.407058913	0.353290139	0.45185911
Cisplatin	0.892018199	0.954891061	0.509208793	0.156328313
Cyclopamine	1.294183296	1.308494941	1.178367609	1.125038784
Digoxin	0.859996134	0.859652727	0.839151724	0.572463941
Doxorubicin	0.001133965	0.000433403	0.000526796	0.000222758
Gentamicin	0.978464623	0.901590061	1.005038328	0.844459295
Itraconazole	0.811276899	0.860434336	0.912661423	0.690312884
LithocholicAcid	0.814940442	0.559973645	0.517536217	0.550848469
MethapyrileneHCl	0.926144406	0.731084319	0.761306772	0.878559273
Thioacetamide	0.457233164	0.152941	0.002991929	0.004797251
ValproicAcid	0.650903022	0.620111817	0.588390887	0.724371395
VerapamilHCl	0.960958908	0.911525752	0.883672711	0.605145708
**Pearson coefficiency**	**1**	**0.945449338**	**0.827102604**	**0.780510906**

Table S6. Growth relative to control for 20 selected chemicals at IC50 concentration of KhES-3 and Pearson correlation between human iPS cell lines and KhES-3, related to Figure 3

Chemical name	Solvent	MaxSolv (ES)	MaxSolv (iPS)	IC0.1	IC50	IC99.9	Exposure 1/1	Exposure 1/2	Exposure 1/4	Exposure 1/8	Exposure 1/16
Acetonylacetone	DMSO	8525	8525	263.4446	3157.525	37844.62	1441.1	720.55	360.275	180.1375	90.06875
Acrylamide	DMSO	10000	10000	329.6103	1244.907	4701.894	915.7	457.85	228.925	114.4625	57.23125
Amiodarone	DMSO	100	100	1.429408	3.321542	7.71833	2.7	1.35	0.675	0.3375	0.16875
AmitriptylineHCl	DMSO	200	200	1.494687	29.03696	564.0949	11.2	5.6	2.8	1.4	0.7
Atorvastatin	DMSO	10	10	0.0554908	2.771251	138.3983	0.7	0.35	0.175	0.0875	0.04375
Axitinib	DMSO	100	100	0.02511065	0.1657731	1.094385	0.08	0.04	0.02	0.01	0.005
Bucillamine	DMSO	1000	1000	14.66608	120.1776	984.7656	61.7	30.85	15.425	7.7125	3.85625
ButylatedHA	DMSO	5000	5000	0.2519234	153.795	93889.21	9.5	4.75	2.375	1.1875	0.59375
Chlorpheniramine	DMSO	1000	2000	20.10651	83.32269	345.2947	52.7	26.35	13.175	6.5875	3.29375
Chlorpromazine	DMSO	300	500	29.18344	75.52795	195.4694	55.6	27.8	13.9	6.95	3.475
Cisplatin	Water	40/0.14	40	0.00112767	1.000171	887.0844	0.07	0.035	0.0175	0.00875	0.004375
Cyclopamine	DMSO	50	50	1.908957	59.27945	1840.824	20.8	10.4	5.2	2.6	1.3
CyclosporinA	DMSO	100	100	0.03722939	1.130822	34.34812	0.3	0.15	0.075	0.0375	0.01875
Digoxin	DMSO	250/0.01	250/0.01	0.00054809	0.04069474	3.021496	0.01	0.005	0.0025	0.00125	0.000625
Doxorubicin	DMSO	3.45	3.45	0.00010333	0.00964146	0.8996372	0.002	0.001	0.0005	0.00025	0.000125
Gentamicin	Water	4500	4500	31.16626	3821.203	468506.3	876.5	438.25	219.125	109.5625	54.78125
Ibuprofen	DMSO	7000	10000	7.387367	214.8406	6248.029	71.2	35.6	17.8	8.9	4.45
Itraconazole	DMSO	18	18	2.02E-06	0.6286174	196023.9	0.06	0.03	0.015	0.0075	0.00375
LithocholicAcid	DMSO	250	250	0.02490118	2.45665	242.3632	0.5	0.25	0.125	0.0625	0.03125
MethapyrileneHCl	Water	2000	2000	20.13058	567.8374	16017.39	169.3	84.65	42.325	21.1625	10.58125
Sunitinib	DMSO	10	10	0.3259165	1.88491	10.90122	1	0.5	0.25	0.125	0.0625
Thioacetamide	Water	2000	2000	19.45127	873.9114	39263.3	251.2	125.6	62.8	31.4	15.7
ValproicAcid	DMSO	6240	6240	85.79245	1989.807	46150.11	640.5	320.25	160.125	80.0625	40.03125
VerapamilHCl	DMSO	500	500	11.78816	67.41076	385.4895	36.3	18.15	9.075	4.5375	2.26875

Table S7. ATP asssay data and exposure concentrations (uM) of HPS4138, related to Figure 3

Chemical name	Estimate	Std. Error	Lower	Upper
Acetonylacetone	3157.525	199.1471	2755.339	3559.711
Acrylamide	1244.907	103.3081	1036.272	1453.542
Amiodarone	3.321542	0.5889884	2.132056	4.511028
AmitriptylineHCl	29.03696	1.427926	26.15321	31.92072
Atorvastatin	2.771251	0.2979128	2.169604	3.372898
Axitinib	0.1657731	0.00723863	0.1511544	0.1803918
Bucillamine	120.1776	6.773494	106.4982	133.8569
ButylatedHA	153.795	40.78266	71.43272	236.1572
Chlorpheniramine	83.32269	3.777015	75.69485	90.95053
Chlorpromazine	75.52795	20.13791	34.85861	116.1973
Cisplatin	1.000171	0.1422687	0.7128536	1.287489
Cyclopamine	59.27945	6.313346	46.52939	72.02952
CyclosporinA	1.130822	0.07621135	0.9769104	1.284734
Digoxin	0.04069474	0.04566175	-0.051521	0.1329105
Doxorubicin	0.00964146	0.00093689	0.00774938	0.01153355
Gentamicin	3821.203	355.5662	3103.122	4539.283
Ibuprofen	214.8406	16.60235	181.3115	248.3697
Itraconazole	0.6286174	0.1412967	0.343263	0.9139718
LithocholicAcid	2.45665	0.2755877	1.900089	3.01321
MethapyrileneHCl	567.8374	36.80672	493.5048	642.1701
Sunitinib	1.88491	0.1394596	1.603266	2.166554
Thioacetamide	873.9114	92.93251	686.2304	1061.592
ValproicAcid	1989.807	201.4727	1582.924	2396.689
VerapamilHCl	67.41076	4.467079	58.38931	76.43221

Table S8. IC50 concentrations (uM) and 95 % confidence intervals of HPS4138, related to Figure 3

Chemical name	24 h	48 h
Acetonylacetone	100	83.3
Acrylamide	83.3	100
Amiodarone	100	100
AmitriptylineHCl	83.3	83.3
Atorvastatin	66.6	100
Axitinib	83.3	83.3
Bucillamine	83.3	83.3
ButylatedHA	50	66.6
Chlorpheniramine	66.6	83.3
Chlorpromazine	83.3	100
Cisplatin	100	83.3
Cyclopamine	100	50
CyclosporinA	83.3	83.3
Digoxin	100	100
Doxorubicin	100	83.3
Gentamicin	83.3	100
Ibuprofen	83.3	100
Itraconazole	83.3	66.6
LithocholicAcid	100	100
MethapyrileneHCl	100	100
Sunitinib	83.3	83.3
Thioacetamide	100	83.3
ValproicAcid	50	100
VerapamilHCl	83.3	50

Table S9. The accuracy of 6 toxicity category predictions for each chemical (%) in HPS4138, related to Figure 3
